# Supplementary material for: Declining Course of Humoral Immune Response in Initially Responding Kidney Transplant Recipients after Repeated SARS-CoV-2 Vaccination
Source: J Clin Med. 2022 Jun 8;11(12):3291. doi: 10.3390/jcm11123291 (PMC9224612; doi:10.3390/jcm11123291)
Supplement: Supplementary file 1 [file jcm-11-03291-s001.zip › jcm-1730868-supplementary.pdf]

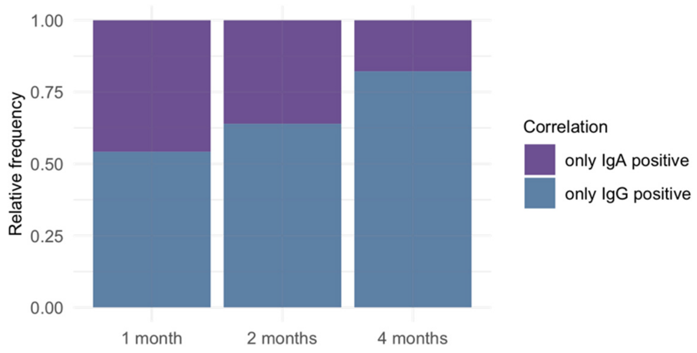

Supplement Figure S1. 266 cases where IgA and IgG assays determined differing results (only IgG positive vs. only IgA positive measurements) and their relative frequency over time.
